# Full-field digital mammography: a retrograde step for small screen-detected cancers?

**DOI:** 10.1186/bcr2950

**Published:** 2011-11-04

**Authors:** P Boavida, E O'Flynn, R Wilson, E O'Donovan, S Allen

**Affiliations:** 1 The Royal Marsden Hospital, London, UK

## Introduction

Our breast surgery service receives many breast cancer referrals from neighbouring breast screening centres with analogue mammography systems. As hospital protocol we perform repeat full-field digital mammography (FFDM) in these women in an attempt to better stage the primary tumours. The aim of this study is to assess whether FFDM detects more disease than analogue mammography in patients with screen-detected cancer with pathological correlation.

## Methods

Three experienced mammography readers evaluated repeat FFDM in 60 women whilst blinded to the prior analogue studies. Sixty age-matched controls were also assessed. The mammographic findings were then compared with the final surgical pathology. Breast density scores were recorded using Quantra™ software to assess whether this influenced diagnostic accuracy.

## Results

Sixteen (26.7%) analogue mammography-detected cancers were not detected by at least one reader on FFDM. Three (5%) analogue mammography-detected cancers were missed by all three readers on FFDM. Small soft tissue masses represented all of the cancers missed by all three readers. Mean fibroglandular density as assessed by Quantra™ was 15.3 for controls, 13.9 for cancers and 14.2 for cancers not identified by at least one reader. FFDM more accurately staged the DCIS extent than analogue mammography when comparing final surgical pathology, although the result was not significant (*P *= 0.2).

## Conclusion

Not all cancers visualised on analogue mammography can be seen on repeat FFDM performed within 2 weeks, even allowing for biopsy-related bruising. Although DCIS is possibly better staged by FFDM compared with analogue, small soft tissue masses are less well distinguished. This effect does not seem related to background breast density.

